# 55-year-old Male with Fatigue

**DOI:** 10.5811/cpcem.2020.11.50651

**Published:** 2021-04-16

**Authors:** Jennifer Nichols, Jennifer Guyther, Laura J. Bontempo, Zachary D.W. Dezman

**Affiliations:** * University of Maryland Medical Center, Department of Emergency Medicine, Baltimore, Maryland; † University of Maryland School of Medicine, Department of Emergency Medicine, Baltimore, Maryland; ‡ University of Maryland, Baltimore, Department of Epidemiology and Public Health, Baltimore, Maryland

**Keywords:** CPC, dialysis, shock

## Abstract

This clinicopathological case presentation from the University of Maryland details the initial assessment and management of a 55-year-old, dialysis-dependent man with fatigue. We present how one of our emergency medicine faculty develops her differential when faced with this complaint. She describes how she arrives at the suspected diagnosis and the test she believes is needed to prove her hypothesis. The final surprising diagnosis is then revealed.

## CASE PRESENTATION (DR. NICHOLS)

A 55-year-old male presented to the emergency department (ED) from his dialysis center with two days of fatigue and generalized weakness. He reported that he had been outside in the heat frequently and admitted to poor fluid intake. He received 250 milliliters (mL) of intravenous (IV) fluid prior to the initiation of dialysis. However, the session was terminated prematurely because the patient began feeling unwell. Emergency medical services (EMS) was called and the patient was transported to the ED. On arrival to the ED, EMS reported that they believed the patient was in supraventricular tachycardia based on their rhythm strip.

The patient’s past medical history was significant for end stage renal disease (ESRD) due to focal segmental glomerulosclerosis, hypertension, secondary hyperparathyroidism, obstructive sleep apnea, chronic pancreatitis, and gastroesophageal reflux disease. He had an arteriovenous fistula placed in his left upper extremity and had a prior left inguinal hernia repair. He was prescribed the following: lidocaine patches, ranitidine, lisinopril, metoprolol succinate, pravastatin, B complex-C-folic acid vitamins, sevelamer carbonate, sildenafil, aspirin, and sodium bicarbonate. He was allergic to chlorhexidine, which resulted in a rash, as well as rosuvastatin, ezetimibe, and atorvastatin, all of which resulted in muscle cramps and an elevation in his serum creatine kinase. He smoked cigarettes (12.5 pack years) and denied using smokeless tobacco, alcohol, or drugs.

On initial examination the patient was awake, alert, well-appearing, and appeared in no acute distress. He was afebrile (36.7º Celcius) with a heart rate of 142 beats per minute (bpm) and a blood pressure of 103/69 millimeters of mercury (mm Hg). He was breathing at a rate of 16 breaths per minute and was saturating at 96% on room air. He weighed 129 kilograms (kg) and was five feet 10 inches tall (1.8 meters) with a body mass index of 41.1 kg/meters^2^. His head was normocephalic and atraumatic. His oropharynx was dry and without erythema or exudate. Extraocular movements were intact. Pupils were equally round and reactive to light. There was no eye discharge. His neck exhibited no jugular venous distension (JVD). His heart sounds were tachycardic but regular and no murmur was auscultated. He had intact distal pulses. He had normal respiratory effort and normal breath sounds, was not in respiratory distress, and had no wheezes. His abdomen was soft and non-tender. He had a left upper extremity fistula with a palpable thrill and audible bruit. There was no extremity edema, tenderness or deformity. He was alert and oriented to person, place, and time. His skin was warm and intact without diaphoresis. He had a normal mood and affect, and his behavior was normal.

**Image 1:**
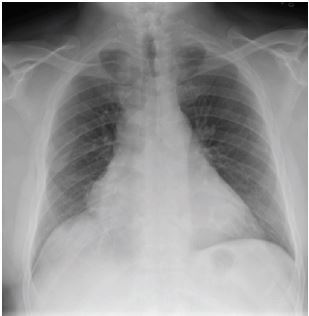
Chest radiograph of a 55-year-old male on hemodialysis who presented to the emergency department with fatigue.

**Image 2:**
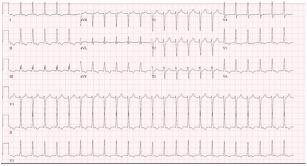
Initial electrocardiogram of a 55-year-old male on hemodialysis who presented to the emergency department with fatigue.

**Image 3:**
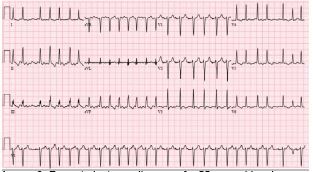
Repeat electrocardiogram of a 55-year-old male on hemodialysis who presented to the emergency department with fatigue.

His initial labs (Table), chest radiograph (CXR) (Image 1) and initial electrocardiogram (ECG) (Image 2) are shown. The patient was initially thought to be in atrial flutter given his heart rate of approximately 150 bpm; however, on repeat ECG, the patient’s heart rate was in the 120s bpm and his rhythm was thought to be most consistent with sinus tachycardia with premature atrial complexes. Therefore, given that the patient also appeared dehydrated, a 500 mL bolus of intravenous fluids (IVF) was given and the patient was observed. His heart rate improved to 100–110s bpm. Two hours later, however, his heart rate increased to 166 bpm and his blood pressure was 135/57 mm Hg. A repeat ECG was obtained (Image 3). At that time, he was thought to be in atrial fibrillation with rapid ventricular response. He was then given metoprolol 5 milligrams (mg) IV and his heart rate improved to 140 bpm.

Shortly thereafter, his heart rate increased to 164 bpm and his blood pressure was 110/58 mm Hg. He was given two 250-mL boluses of IVF and three doses of metoprolol 5 mg IV, five minutes apart. His heart rate improved to 98 bpm and his blood pressure decreased to 94/58 mm Hg. He appeared to be in normal sinus rhythm after these interventions. The patient then reported chest tightness and was given a dose of aspirin 324 mg. About one hour later, he became hypotensive (76/60 mm Hg) with a heart rate of 148 bpm. His rhythm was interpreted as atrial fibrillation with rapid ventricular response. He was sedated with ketamine and cardioverted using a 100-joule biphasic shock, which resulted in conversion to sinus tachycardia with a rate of 105 bpm and blood pressure of 115/78 mm Hg. Subsequently, the patient was started on a heparin infusion. He had two serial troponins obtained, both of which were within normal limits (0.04 nanograms (ng)/mL and lastly 0.05 ng/mL).

While boarding in the ED awaiting admission to cardiology, the patient was reassessed three hours later and was found to be dizzy, diaphoretic, and pale, with a heart rate of 159 bpm and blood pressure of 92/55 mm Hg. An ECG was obtained and read as atrial fibrillation with rapid ventricular response. He was given metoprolol 5 mg IV, metoprolol 12.5 mg orally, and an amiodarone bolus of 300mg IV followed by an infusion. Then a test was performed, and a diagnosis was made.

## CASE DISCUSSION (DR. GUYTHER)

Based on the first few lines of the patient presentation, this case seems simple and straightforward: a middle-aged man with ESRD who was not feeling well and was dehydrated due to heat exposure, who was given IV fluids. He then develops unstable vital signs and decompensates while in the ED. Looking at the patient’s chief complaint (fatigue and weakness), I feel fatigued myself. The combined differential of weakness, fatigue, and ESRD is overwhelming. I decided to look at the individual clues to narrow down and help me solve the puzzle of this patient’s presentation.

The history is supposed to lead you to the diagnosis in most cases. Here our history of present illness is unhelpful. The patient went to dialysis, suggesting that he is compliant with his medications and medical treatments. He received a small bolus of IVF prior to dialysis initiation, which seems logical in the setting of poor oral intake and being outside in the heat, but he was unable to tolerate his full session of dialysis because he did not “feel well.” At this point in the case, I am not sure what to do with that last piece of information, but I am concerned that such a small amount of fluid would make the patient feel he needs to stop his dialysis.

He seems to be on appropriate medications for his history of ESRD, including three antihypertensive medications. I did not gain any additional clues from his past surgical history, allergies, family history or social history. His extensive review of systems also did not add any helpful information.

Moving to the physical exam, his dry lips suggest low intravascular volume. His vital signs are significant for tachycardia to 142 bpm and, for this patient with a history of hypertension, a likely relative hypotensive blood pressure of 103/69 mm Hg. He is not in acute distress and he is alert and oriented to person, place and time, suggesting he isn’t in shock due to these vital signs. It is also important to note that he has no fever, tachypnea, or hypoxia.

The results of his blood testing are consistent with what would be expected for a dialysis-dependent patient: mild anemia and thrombocytopenia, as well as an elevated blood urea nitrogen and creatinine. His troponin was normal. His CXR shows cardiomegaly, which would also be expected in patients with ESRD. The first ECG showed a rate of about 150 bpm with visible p-waves and t-wave inversions in the inferior and lateral leads. The patient was given a 500 mL bolus of IVF with improvement of his heart rate to 110 bpm. About three hours later the patient’s heart rate was in the 160s bpm and his new ECG showed atrial flutter with variable conduction. His heart rate remained elevated and he became increasingly hypotensive despite multiple doses of beta blockers and fluid boluses. The patient then went into atrial fibrillation with a rapid ventricular response and required cardioversion in the setting of persistent hypotension. Looking through his history and medications, I found he did not have any known problems with dysrhythmias. The patient experienced some chest tightness and while his troponins increased slightly, I would expect an elevation after a tachydysrhythmic episode and a cardioversion.

Overall, there are two large pieces of this puzzle: ESRD and atrial tachydysrhythmias. I find it easier to focus first on the differential for fast heart rates compared to the complaints of dizziness and fatigue.

The patient was reported to be in supraventricular tachycardia by EMS. The initial ECG done in the ED showed concern for atrial flutter with variable conduction. The causes of atrial flutter include myocardial ischemia (MI), hypoxia, congestive heart failure (CHF), chronic obstructive pulmonary disease (COPD), pericarditis, and hyperthyroidism. Anything that causes irritation of the myocardium could cause a dysrhythmia. Myocardial ischemia and CHF should remain on the differential based on the patient’s risk factors and CXR findings. The patient was never hypoxic and although he does have a 12.5 pack year history of smoking, he has no pulmonary or CXR findings to support the diagnosis of COPD, so this can be removed from the list. The patient does not have clinical manifestations of hyperthyroidism, aside from the tachycardia, and I would not have expected such a drastic decline from this pathology alone while in the ED.

When I cross-referenced this differential diagnosis of atrial flutter with the causes of atrial fibrillation, the diagnoses of CHF, cardiomyopathy, MI, and pericarditis remain high on the differential for this patient. Endocarditis can remain on the differential as the patient is at risk for bacteremia given his intermittent dialysis access and his elevated white blood cell count. Acquired or native cardiac valvular disease, like atrial septal defects and mitral stenosis, as well as the procedures needed to correct these defects, can cause atrial fibrillation, but these are not supported by the patient’s past medical history and exam. Noncardiac causes for atrial fibrillation include pulmonary embolism, pneumonia, and hypoxia, as well as alcohol or cocaine use, sepsis, and hyperthyroidism. His social history does not support substance abuse. I would expect the patient to have some hypoxia or shortness of breath with a massive pulmonary embolism, so this condition falls much lower down on my differential diagnosis.

I used the cardiac complications of ESRD to further refine my differential of MI, CHF, and endocarditis and less likely hyperthyroidism. The cardiac complications of ESRD include coronary artery disease, CHF, and pericardial effusion due to uremia. Although the patient was experiencing chest tightness and did have some ECG changes, his troponins did not significantly elevate and repeat ECGs did not show ST changes, so the patient probably wasn’t taken to the cardiac catheterization lab to diagnose an MI. The patient likely has some underlying component of CHF, but he doesn’t have the findings on physical exam or CXR to suggest a hypervolemia severe enough to cause a dysrhythmia. The patient is uremic on his labs, so he may have a uremic pericardial effusion.

Taking everything into account, especially how the patient decompensated after starting the heparin drip, my highest concern would be for a uremic pericardial effusion that has now converted into a hemorrhagic effusion with tamponade. An echocardiogram would confirm this diagnosis.

## CASE OUTCOME (DR. NICHOLS)

The diagnostic test was an echocardiogram that demonstrated a pericardial effusion and cardiac tamponade. The patient underwent emergency pericardiocentesis with drain placement in the cardiac catheterization lab; 960 mL of dark red fluid was drained with subsequent improvement in the patient’s hemodynamics. He was discharged on post-operative day 4.

## RESIDENT DISCUSSION (DR. NICHOLS)

Cardiac tamponade is defined as compression of all cardiac chambers resulting in a reduction of venous return and thus a decrease in cardiac output.^1^ There are four types: acute; subacute; low pressure; and regional. Acute cardiac tamponade presents with the classic symptoms of chest pain and dyspnea as well as the classic exam findings, which are Beck’s triad (hypotension, JVD, and muffled heart sounds), tachycardia, and pulsus paradoxus. Tachycardia is a physiological compensation to offset the loss in cardiac output due to a decrease in stroke volume. Pulsus paradoxus is defined as a 10 mm Hg decrease in systolic pressure on inspiration and occurs because of reduction of left heart filling on inspiration.^2^ Subacute tamponade is generally more insidious, with patients presenting with nonspecific symptoms such as chest discomfort or fullness, dyspnea, peripheral edema, or feeling easily fatigued. Low pressure occurs in patients who are severely hypovolemic (for example, those who experienced a traumatic hemorrhage, undergo hemodialysis, or are over-diuresed).^1^ Giving these patients an IVF challenge will actually elicit tamponade pathophysiology due to the increase in fluids in the pericardial sac. Regional tamponade occurs when there is a loculated or eccentric effusion or localized hematoma. It is important to emphasize that patients with low pressure or regional tamponade do not typically present with the classic physical exam, hemodynamic, and echocardiographic findings.

Etiologies of pericardial effusions are idiopathic, infectious, autoimmune/inflammatory, neoplastic, cardiac (post-cardiac injury [Dressler’s syndrome], myocarditis, dissecting aortic aneurysm, early infarction pericarditis), traumatic, metabolic (hypothyroidism, uremia, ovarian hyperstimulation syndrome), or drug-induced (procainamide, isoniazid, and hydralazine). Idiopathic, infectious, and neoplastic causes are most common.^3^ Findings on ECG include the following: tachycardia; low voltage, electrical alternans (beat-to-beat changes in QRS amplitude or axis); and evidence of pericarditis (diffuse ST elevations, PR depressions, down-sloping TP segments).^1^

Echocardiography can be used to identify the pericardial effusion and evaluate for signs of tamponade.^4^ These signs include the following: chamber collapse (diastolic collapse of the right atrium and ventricle as well as left-sided chamber collapse)^4^,^5^; respiratory variations in volumes and flows (increased flow across mitral and tricuspid valves on inspiration)^6^; and inferior vena cava (IVC) dilation and decreased collapsibility.^7^ Right atrial collapse is highly sensitive and specific (100% and 82%, respectively).^8^ Right ventricular collapse is not as sensitive as it may be absent in patients with increased right ventricular pressure (for example, pulmonary hypertension, right ventricular hypertrophy, constrictive pericardial disease, etc), but it is very specific (82% and 90%, respectively).^9^ Left-sided collapse occurs only in about 25% of patients because of how muscular the left side of the heart is, but it is frequently found in patients with regional cardiac tamponade.^10^,^11^

Cardiac tamponade patients should be treated with emergent drainage of the effusion.^12^ This can be accomplished at the bedside or in the cardiac catheterization laboratory by pericardiocentesis (either blind or ultrasound-guided) with or without catheter placement for continued drainage. If patients are stable, they can be taken to the operating room for open surgical drainage with or without pericardiectomy (pericardial window) or video-assisted thoracoscopic pericardiectomy.^13^ Surgical drainage may be preferable in patients with small effusions, loculated effusions, aortic dissection, myocardial rupture, or those requiring biopsies.^4^

## FINAL DIAGNOSIS

Cardiac tamponade

**KEY TEACHING POINTS**

- Shortness of breath, chest pain, or fatigue in a dialysis (or cancer) patient should prompt a point-of-care ultrasound to evaluate for a pericardial effusion.
- Classic exam findings of cardiac tamponade include the following: Beck’s triad (hypotension, JVD, and muffled heart sounds), as well as tachycardia and pulsus paradoxus, but these findings may not be present in patients with low blood pressure or regional tamponade.
- Echocardiographic findings suggestive of cardiac tamponade include right atrial/ventricular and left heart collapse, increased flow across the mitral and tricuspid valve on inspiration, and a plethoric IVC.

## Figures and Tables

**Table:** Laboratory test results of a 55-year-old male on hemodialysis who presented to the emergency department with fatigue.

Laboratory test	Result	Normal range
Complete blood count		
White blood count	14.3 K/mcL	4.5–11 K/mcL
Hemoglobin	9.8 g/dL	12.6–17.4 g/dL
Hematocrit	30.3%	37–50%
Platelets	142 K/mcL	153–367 K/mcL
Chemistry		
Sodium	137 mmol/L	136–145 mmol/L
Potassium	4.6 mmol/L	3.5–5.1 mmol/L
Chloride	95 mmol/L	98–107 mmol/L
Bicarbonate	25 mmol/L	21–30 mmol/L
Anion gap	17	4–16
Blood Urea Nitrogen	51 mg/dL	9–20 mg/dL
Creatinine	17.05 mg/dL	0.66–1.25 mg/dL
Glucose	147 mg/dL	70–99 mg/dL
Calcium	9.7 mg/dL	8.6–10.2 mg/dL
Magnesium	2.7 mg/dL	1.6–2.6 mg/dL
Total Protein	7.8 g/dL	6.3–8.2 g/dL
Albumin	4.3 g/dL	3.5–5.2 g/dL
Aspartate Transaminase	22 units/L	17–59 units/L
Alanine Transaminase	25 units/L	0–49 units/L
Bilirubin Total	0.7 mg/dL	0.3–1.2 mg/dL
Alkaline Phosphatase	64 units/L	38–126 units/L
Cardiac Troponin I	0.02 ng/mL	<0.06 ng/mL

*K*, thousand; *mcL*, microliter; *g*, grams; *dL*, deciliter; *mmol*, millimole; *L*, liter; *mg*, milligram.
